# Mechanical compression insults induce nanoscale changes of membrane-skeleton arrangement which could cause apoptosis and necrosis in dorsal root ganglion neurons

**DOI:** 10.1080/09168451.2014.932664

**Published:** 2014-07-14

**Authors:** Xin Quan, Kai Guo, Yuqing Wang, Liangliang Huang, Beiyu Chen, Zhengxu Ye, Zhuojing Luo

**Affiliations:** ^a^ Department of Orthopedics, Xijing Hospital, The Fourth Military Medical University, Xi’an, The People’s Republic of China

**Keywords:** mechanical compression, spinal cord injury, membrane-skeleton, apoptosis, atomic force microscopy

## Abstract

In a primary spinal cord injury, the amount of mechanical compression insult that the neurons experience is one of the most critical factors in determining the extent of the injury. The ultrastructural changes that neurons undergo when subjected to mechanical compression are largely unknown. In the present study, using a compression-driven instrument that can simulate mechanical compression insult, we applied mechanical compression stimulation at 0.3, 0.5, and 0.7 MPa to dorsal root ganglion (DRG) neurons for 10 min. Combined with atomic force microscopy, we investigated nanoscale changes in the membrane-skeleton, cytoskeleton alterations, and apoptosis induced by mechanical compression injury. The results indicated that mechanical compression injury leads to rearrangement of the membrane-skeleton compared with the control group. In addition, mechanical compression stimulation induced apoptosis and necrosis and also changed the distribution of the cytoskeleton in DRG neurons. Thus, the membrane-skeleton may play an important role in the response to mechanical insults in DRG neurons. Moreover, sudden insults caused by high mechanical compression, which is most likely conducted by the membrane-skeleton, may induce necrosis, apoptosis, and cytoskeletal alterations.

## Graphical Abstract

Mechanical compression can cause rearrangement of the membrane skeleton and cytoskeleton and different biological responses of DRG neurons.

**Figure:**
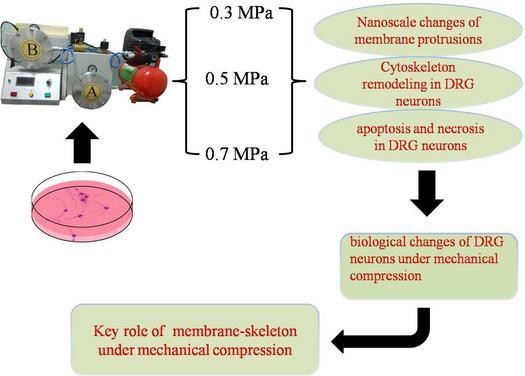

The incidence of traumatic spinal cord injury (SCI) has presented an increasing trend over the last few decades.^1^ ^)^ SCI involves an acute phase of primary injury that causes immediate cell damage and vascular disruption followed by a phase of delayed secondary injury that may last from hours to weeks.^2^ ^)^ The primary injury usually involves direct compression of the spinal cord as a result of dislocation of the spinal vertebral motion segments or displaced bone fragments. Morphological characteristics and clinical outcomes vary depending on the force of the spinal cord compression, duration of compression, displacement of the cord, acceleration of impacting forces, and kinetic energy absorbed at the time of spinal cord compression.^3,4^ ^)^ Therefore, mechanical compression is one of the most critical primary injury factors involved in SCI. Almost all SCI occurring in humans are due to compression of the spinal cord. Therefore, simulating this mechanical compression is necessary to study SCI, and a number of experimental models have been developed to simulate acute clinical mechanical compression of the spinal cord.

Previous experimental models have demonstrated that spinal cord tissue responds to weighted loads in a predictable manner that can be mathematically modeled under precise loading conditions.^5–7^ ^)^ Preferably, *in vitro* models are used to study the events immediately after the application of a trauma stimulus as they allow precise control over the extracellular environment and are easily accessed, highly reproducible, and lower in cost.^8^ ^)^ In addition, *in vitro* models, by virtue, reduce animal pain resulting from *in vivo* experiment and thus eliminate the need for post-operative animal care procedures. 

The formation of plasma membranes (PMs) is most likely a crucial event during evolution, and their integrity is important for the maintenance of normal cellular function.^9^ ^)^ Given their critical role, membranes constitute a cellular Achilles heel, sensitive to both mechanical rupture and molecule-driven alterations.^10^ ^)^ The membrane skeleton proteins have been reported to be involved in many physiological and pathological processes, including joint function restoration,^11^ ^)^ mechanical deformation disease,^12^ ^)^ and maintenance of axonal transportation.^13^ ^)^ In the primary SCI phase, neuronal cell membranes demonstrate a critical role in the reaction to mechanical compression, which likely results in alterations of cell morphology, cell vitality, gene expression, and cytoskeletal organization. Although biochemical assays are useful in revealing the level of these changes,^14^ ^)^ ultrastructural studies can provide unique information about the features of cell-membrane skeletal proteins. However, there are limited studies on the nanoscale changes of the superficial topography of neuronal cell membranes in reaction to mechanical compression forces that simulate primary SCI.

Atomic force microscopy (AFM) has emerged as a powerful analytical tool in medical, biological, and biophysical research due to its unique abilities. AFM reveals novel information about the membrane structure, cell organelles, and the cytoskeleton at a molecular resolution.^15^ ^)^ In addition, AFM can provide quantitative descriptions of the morphological details of biomolecular distributions and arrangements under different conditions. In recent years, it has been used to probe interaction forces through a mode known as force spectroscopy.^16^ ^)^ It is widely used for variety of applications to measure surface interaction forces. These functions serve as a valuable tool to elucidate ultrastructural changes in cell-surface topography at the nanoscale level and to observe changes in the distribution of cell-surface molecules that associate with biochemical or biomechanical signal-induced biological results such as apoptosis. This information may provide novel insights into the control of cell shape and interactions as a result of mechanical compression stimulation. AFM can also serve as a valuable tool to provide insights into the resulting changes in the cell, such as its mechanical properties, membrane tension, and mechanical sensitivity.

In our present study, to investigate the effects of mechanical pressure on neurons at the ultrastructural level, we have examined changes in the surface topography of dorsal root ganglion (DRG) neurons and the occurrence of cell apoptosis and necrosis in response to mechanical stress. Using AFM, we investigated changes in the topological details of cultured DRG neurons following the application of mechanical compression forces. We hypothesized that changes in the membrane particle structure accompany changes in mechanical force. Additionally, the mechanical compression force may lead to apoptosis and necrosis caused by remodeling of the membrane-skeleton of neurons, which is relevant to the mechanical compression intensity. Our purposes were as follows: (1) to elucidate qualitative and quantitative changes in the surface topology of DRG neurons under the above conditions, (2) to explore accompanying cytoskeletal remodeling, and (3) to investigate the influence of mechanical forces on cell survival. These studies would provide novel insights into the physical and morphological properties of DRG neurons following mechanical stimulation and would be useful for searching for further mechanisms of and protective agents for mechanical compression injury.

## Materials and methods

#### Cell culture

All animals were obtained from the Animal Centre of the Fourth Military Medical University, and procedures were approved by the administration of the Committee of Experimental Animals in Shaanxi, China. Primary cultures of DRG neurons were prepared as previously described.^17^ ^)^ Briefly, newborn Sprague Dawley rats were submerged in 95% ethanol for 2 min. DRGs were aseptically removed and saved along with as many rootlets as possible. The tissue was finely minced with eye scissors and digested in a mixture containing 0.1% collagenase and 0.25% trypsin (Invitrogen, Carlsbad, CA, USA) for 25 min at 37 °C. The cell suspension was then centrifuged at low speed for 4 min, and the supernatant was discarded. The DRG neurons were then gently washed three times for 5 min each in 5 mL of Dulbecco’s Modified Eagle Medium (Invitrogen, USA) with 10% fetal bovine serum (Hyclone, Logan, UT, USA). The cell pellet was resuspended in Neurobasal Medium (Gibco^®^, Grand Island, New York State, USA) consisting of 1% B-27, 10 ng/mL nerve growth factor, 2500 mg/mL glucose, and 2 g/LNaHCO_3_. The cells were seeded onto cover slips or culture plates precoated with poly-L-lysine and laminin. The cultures were incubated at 37 °C in a humidified atmosphere of 5% CO_2_/95% air, and the medium was replaced routinely every 2 days.

#### Mechanical injury model and experimental design

We used a self-designed, mechanical pressure-controlled cellular injury unit, which was described in detail in our previous study.^14^ ^)^ Briefly, this unit consists of three parts: the mechanical pressure container, the pressure control system, and the gas booster.^14^ ^)^ The unit can precisely simulate mechanical pressure injuries *in vitro* while excluding other confounding factors. Based on our previous study, mechanical pressure was set at 0.3, 0.5, and 0.7 MPa, in addition to a negative control for 10 min in the main experiment. The cultured DRG neurons were divided into four treatment groups as follows: control, 0.3 MPa of compression, 0.5 MPa of compression, and 0.7 MPa of compression. All cells were cultured immediately after injury for 24 h before subsequent experimental steps.

#### Quantitative determination of necrosis using Flow Cytometry

The necrosis produced by the effects of mechanical compression was detected by the Propidium Iodide kit (Biotium, Inc, Hayward, CA, USA) by using Flow Cytometry. DRG neurons from each group were harvested with 0.125% trypsin and fixed with 75% (v/v) ethanol overnight at 4 °C. The cells were then resuspended and incubated with 100 mg/mL RNase A at 37 °C for 30 min. Cell nuclei were stained with 50 mg/mL PI for an additional 30 min. Cells were stained according to the kit’s protocol, and were analyzed by a Flow Cytometer (BD Biosciences, San Jose, CA, USA). The determinations were performed in duplicates.

#### Detection of caspase-3 activity

The activity of caspase-3 was measured using a spectrophotometric assay kit for mammals (KeyGEN BioTECH, Nanjing, CHN) following the manufacturer’s instructions. Briefly, 3 × 10^6^ cells per group were pelleted by centrifugation (1000 rpm, 5 min). The cells were resuspended in 50 μL of cell lysis buffer (as supplied by the manufacturer) and incubated for 20 min on ice. The insoluble fraction was discarded by centrifugation (10,000 rpm, 1 min, and 4 °C), and the protein content in the supernatant was determined with the Bradford method. The supernatant(containing 100–200 μg protein) of each sample was diluted with the cell lysis buffer to 50 μL and the diluent was mixed with 50 μL 2× reaction buffer to make a 100 μL reaction mixture. Five microliter corresponding substrate was added to the reaction mixture of each sample to determine the activity of caspase-3. After incubation of the assay mixture for 4 h at 37 °C in the dark, the released paranitroaniline was measured at 405 nm using a Synergy HT multi-well plate reader (Scientific Instruments, Shanghai, CHN). Caspase activity was expressed as relative fluorescence intensity.

#### Transmission electron microscope

The samples for TEM were prepared as follows. Briefly, after being incubated post mechanical compression, 1 × 10^6^ cells were harvested for each group and then the cells were fixed with 2.5% glutaraldehyde (Sigma–Aldrich, USA) in 0.1 M phosphate-buffered saline solution (PBS) overnight at 4 °C. The cells were washed with PBS twice and postfixed with 1% osmium tetroxide (Sigma–Aldrich, USA) in PBS for 45 min at 4 °C. Then the cells were dehydrated in graded series of ethanol (Sangon, CHN), starting at 50% each step for 5 min, after two changes in propylene oxide (Sigma–Aldrich, USA). The tissue specimens were embedded in Araldite (Sigma–Aldrich, USA). Ultrathin section was stained with Mg-uranyl acetate and lead citrate (Sigma–Aldrich, USA) for TEM evaluation.

#### Atomic force microscopy (AFM) observation

AFM imaging was performed using a Multimode AFM instrument (Veeco, Santa Barbara, CA, USA) equipped with a Nanoscope IIIa controller. After mechanical compression, DRG neurons on poly-lysine-treated cover glass were fixed with paraformaldehyde for 20 min, and then a drop of distilled water was added to wash and remove sodium chloride from the sample as sodium chloride will crystallize after the sample is dried and disturb the observation. Subsequently, most of the solution was removed by filter paper and air drying. The glass coverslip with attached cells was glued to a steel disk and placed on the stage of the AFM. Then, the specimen was imaged in Tapping Mode in the air at room temperature. The sample was scanned under a sharpened pyramidal tip (silicon nitride probes, Veeco, Santa Barbara, CA, USA) with a diameter of 20 nm and a spring constant of 0.32 N/m. Images were acquired at 512 lines per scan direction using a scan rate of 0.8–1.5 Hz. Images were recorded for the height and amplitude channels. The vertical height of the cell-surface features was estimated using the “Nanoscope analysis” tool of the Nanoscope software on the AFM height images. At least three independent specimens in each experimental condition were observed under AFM, and data were collected from at least 20 different cells for each sample. At least six different regions from each AFM image, each encompassing fifty protrusions on the cell-surface, were analyzed for each sample to estimate the sizes of granular structures. The statistical analysis of the particle height was weighted using one-way analysis of variance (ANOVA) with samples considered correlated and significance at *p *< 0.05.

#### Immunohistochemistry staining

The purity of the DRG neurons was analyzed with immunohistochemistry. After being grown on cover slips, the cells were then fixed with 4% paraformaldehyde (Haide Bio, China) in phosphate buffer solution (PBS) for 25 min at room temperature, treated with 1% hydrogen peroxide (Zhongshan Bio, China) for 10 min, and incubated for 40 min with blocking solution (1% bovine serum albumin [BSA], 0.4% Triton X-100, and 4% normal serum in PBS) (Cellgro^®^, USA). The cells were incubated with primary antibodies (1:1000) (Abcam, UK) overnight at 4 °C and then with fluorescein isothiocyanate-conjugated rabbit anti-mouse IgG (1:50) secondary antibodies (Abcam, UK) at 37 °C in the dark. The cells were then stained with a 100 ng/mL solution of 4’, 6-diamidino-2-phenylindole (DAPI) for 5 min in the dark. Between each step, the cells were rinsed extensively three times for 5 min each. The stained cells were viewed with a fluorescent microscope (Olympus, Japan) by an observer blinded to the sample conditions.

#### Coomassie brilliant blue (CBB) staining

The cytoskeletal arrangement was evaluated by CBB staining as described in a previous study.^18^ ^)^ Briefly, at the end of the experimental procedure, cover slips with cells from each mechanical compression group were fixed with 4% paraformaldehyde (Haide Bio, China) in phosphate-buffered saline (pH 7.4) for 20 min at 4 °C. Then, the cover slips were treated with 10% Triton-X100 (Zhongshan Bio, China) for 5 min, and the CBB staining solution was added to label the cytoskeleton for 30 min. All incubation steps were performed at room temperature, and between each step, the cells were rinsed extensively three times for 5 min. After staining, the cover slips were dipped into the differentiation solution for a short time to enhance the cytoskeletal staining.

#### Detection of cell apoptosis by transferase-mediated nick end labeling staining

The apoptosis levels of the DRG neurons were assessed by *in situ* terminal deoxynucleotidyl transferase-mediated nick end labeling (TUNEL) staining (Roche, Germany). The experimental procedures were performed according to the manufacturer’s protocol. Briefly, the cover slips were washed with 0.9% PBS, fixed with 4% paraformaldehyde, and washed three times with PBS by rinsing the slides in PBS and holding them in PBS for 2 min between washes. The slides were then subjected to the TUNEL-ApopTag Plus fluoresce *in situ* apoptosis assay (Roche, Germany) following the manufacturer’s instructions. Then, the cover slips were stained with a 100 ng/mL solution of DAPI for 5 min in the dark. The slides were mounted as described above and stored at 4 °C in the dark until viewed. The percentage of apoptotic neurons from six randomly selected fields was evaluated for each experimental group by counting the total number of TUNEL-positive cells. Cells that showed co-localization of both the TUNEL signal and DAPI were considered to be TUNEL-positive cells. All counts were made by viewing slides under a fixed magnification of 40× using a laser confocal microscope (Olympus, Japan).

#### Statistical analyses

All values are expressed as the means ± SEM. The statistical analyses were performed by one-way ANOVA. A *p*-value of less than 0.05 was considered to be statistically significant.

## Results

### Differences in the distribution of small membrane protrusions and larger uplifted particles among different mechanical compression groups

**Fig. 1.**
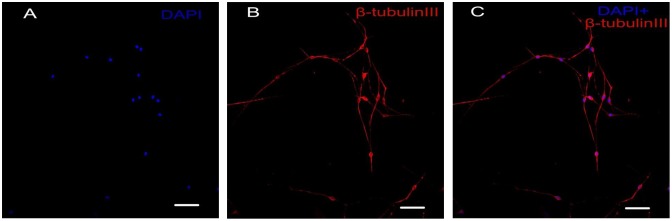
Immunocytochemistry staining of cultured primary DRG neurons. Notes: The DRG neurons were stained with neuronal specific antibody β-tubulin III(B, red), the nucleus were stained with DAPI (A, blue), and the double label is shown in (C) Scale bar, 50 μm.

**Fig. 2.**
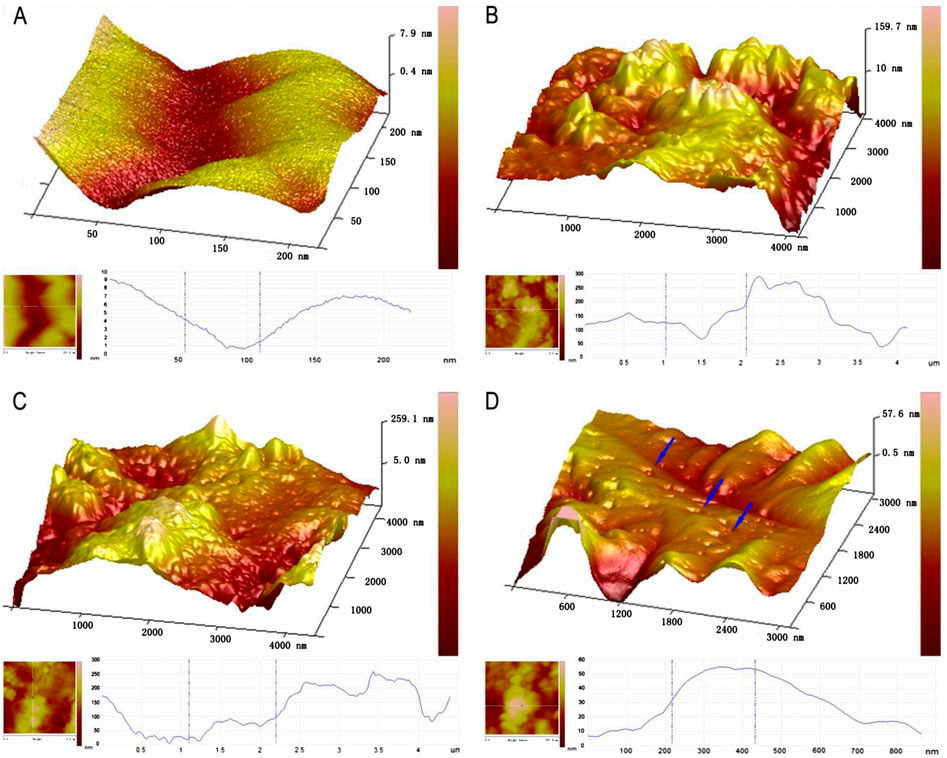
AFM amplitude images of the membrane of native DRG neurons (A) and those subjected to different mechanical compression (B–D) for 10 min with subsequent culture for 24 h as indicated. Notes: The surface morphology of stimulated cells has more granular particles as compared to unstimulated cells. Remodeling of the membrane skeleton can be observed in mechanical compression treated cells as compared to their untreated controls. The smaller pictures on the bottom of each large picture show a quantitative vertical profile along a white line across the AFM image (bottom of each picture, the left), which enables measurement of the height of granular structures. A, the control group; (B) 0.3 MPa group; (C) 0.5 MPa group; and (D) 0.7 MPa group. Blue arrows in (D) show the representative “mechanoporation phenomena” structure in cell membrane in 0.7 MPa group. Scale bar was presented in each photograph.

**Fig. 3.**
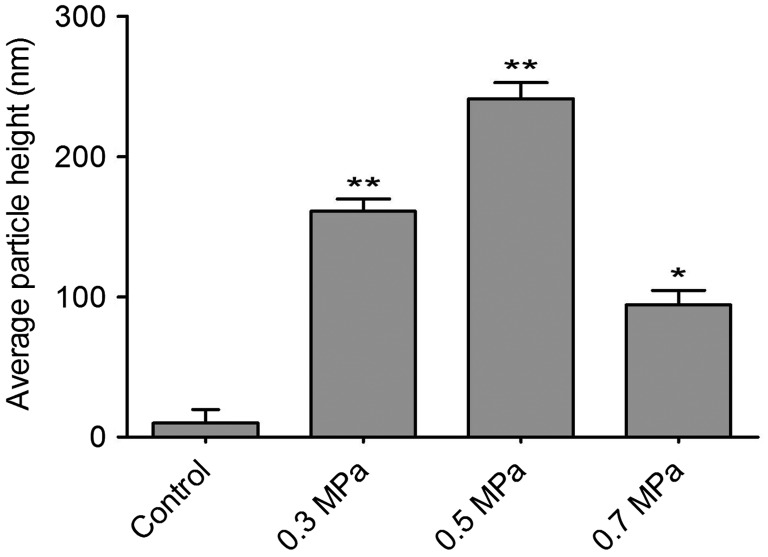
Quantification of cell-surface granules ascertained from AFM images for various cell samples as indicated in Fig. 2. Notes: The bar graph represents the mean height (±SEM) of particles in each condition. Samples with mechanical compression value in 0.3, 0.5, and 0.7 MPa group have a significant higher average particle size than their untreated controls (*p < 0.01, **p < 0.001).

The cultured DRGs were stained with a neuronal-specific antibody, β-tubulin III, and the purity of the cultured DRG neuron cells was 95 ± 2.3% (Fig. 1). The topography of the cell-surface local areas changed tremendously among the different mechanical compression groups. Compared with the control group, which was subjected to the same experimental procedures except the mechanical stimulation, the neuronal membrane ultrastructure of the experimental group presented obvious differences. In the control group, a relatively smooth surface was presented on the neuronal cells, and very few small protrusions above the height of 50 nm were found (Fig. 2(A)). However, many small membrane protrusions and larger uplifted particles were observed in the experimental groups. The average height of the membrane protrusions in the 0.3 and 0.5 MPa groups (Fig. 2(B) and (C)) correlated with the mechanical value, indicating that the membrane skeleton responds actively to mechanical stimulation. Quantitative analysis (Fig. 3) showed that the average heights of the relatively large uplifted structures above the cell surface for 50 granules were significantly increased (*p *< 0.001) in the 0.3 MPa (161.000 ± 8.765 nm) and 0.5 MPa (241.167 ± 11.472 nm) groups compared with the control group (10.167 ± 9.457 nm). However, the higher value of mechanical compression in the 0.7 MPa group (94.167 ± 10.387 nm) (Fig. 2(D)) did not induce a rougher membrane surface than those of the 0.3 and 0.5 MPa groups but was still significantly increased (*p *< 0.01) compared with that of the control group. Nevertheless, the membrane in the 0.7 MPa group presented some “pore canal” structures (Fig. 2(D)). In addition, three-dimensional pictures showed that the topography of the membrane surface was not rougher than that of the 0.3 and 0.5 MPa groups (Fig. 2(D)).

### Mechanical compression leads to cytoskeleton remodeling in DRG neurons

**Fig. 4.**
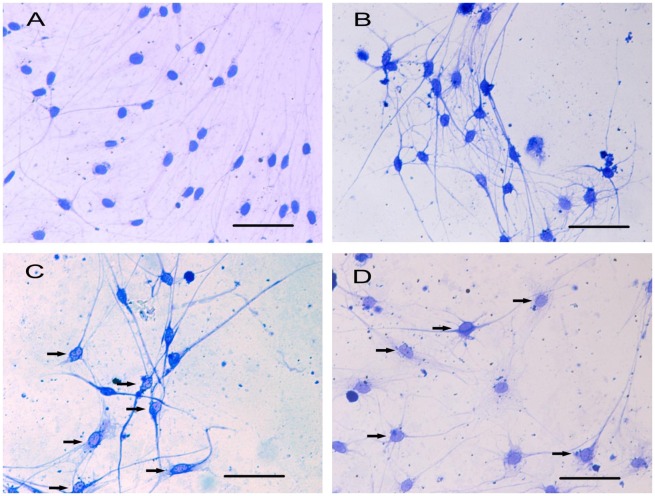
CBB staining shows different distribution of cytoskeleton in cultured DRG neurons. Notes: The distribution of cytoskeleton is nearly normal and there are no significant marginalized gathered cytoskeleton in DRG neurons in control group (A) and 0.3 MPa group (B). However, in 0.5 MPa group (C) and 0.7 MPa group (D), the distribution of cytoskeleton changed obviously, with some nonuniformed distribution of cytoskeleton. Black arrows show the marginalized gathered cytoskeleton in mechanical-treated cells. Scale bar, 50 μm.

CBB staining showed that the cytoskeletal arrangement in the DRG neurons was modified in response to mechanical compression. Evident remodeling of the cytoskeleton could be found in the 0.5 and 0.7 MPa groups, with marginalized gathered cytoskeleton distributed around the cell membrane and a decreased distribution of the cytoskeleton in the cell body (Fig. 4(C)–(D), black arrow). In the control group, the CBB-stained cytoskeleton was distributed homogeneously around the cell body and within the neurites (Fig. 4(A)). The results of the AFM observation showed that the cortical skeleton on the cell membrane was also modified in response to mechanical compression, which is consistent with the CBB staining results.

Immunohistochemistry staining showed the similar distribution of microtubulin-III with CBB staining under the mechanical compression (Supplemental Fig. 1, see *Biosci. Biotechnol. Biochem.,* Web Site.). Marginalized gathered cytoskeleton in DRG neurons can be found in mechanical compression group. In addition, we can find retraction, fracture, or collapsing of β-tubulin III by immunocytochemistry staining in different mechanical compression groups.

### Mechanical compression induces apoptosis and necrosis in DRG neurons

**Fig. 5.**
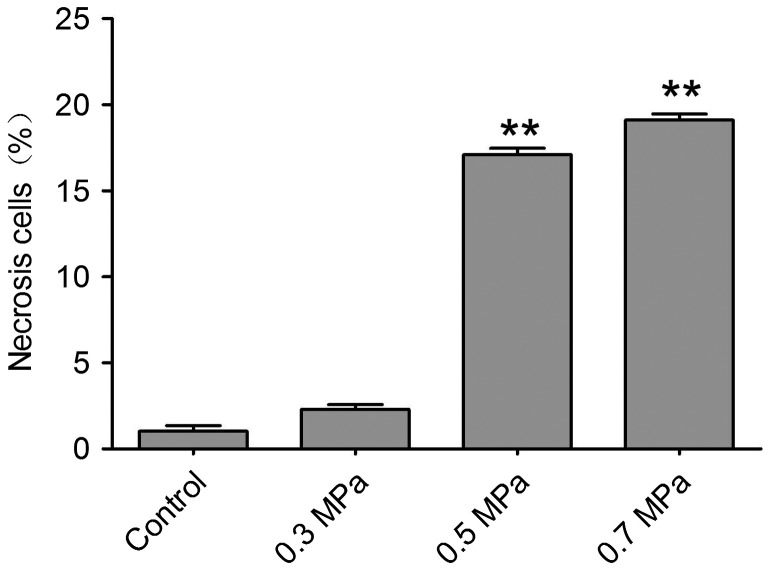
Quantitative determination of necrosis using Flow Cytometry. Notes: Cell necrosis can be found by using Flow Cytometry in different mechanical compression. The bar graph represents the mean percentage (±SEM) of necrosis cells in each condition. In the mechanical compression groups, except 0.3 MPa group, the percentage of necrosis cells increased significantly compared with the control group (**p < 0.01).

**Fig. 6.**
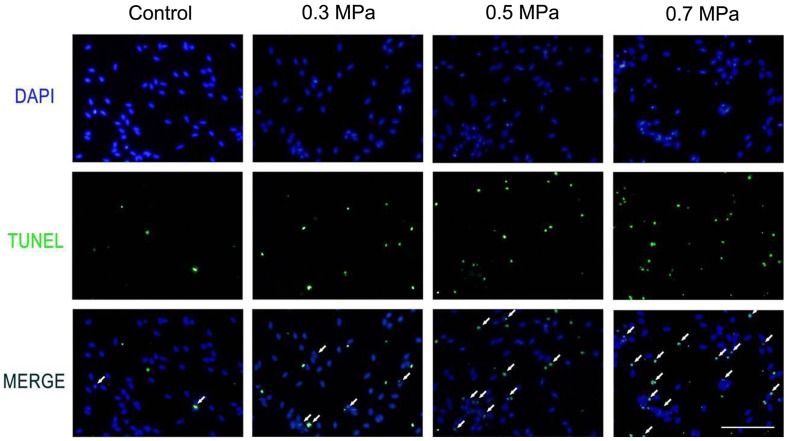
TUNEL detection show different scale of cell apoptosis in different mechanical compression groups. Notes: Compared with the control group (A–C), the mechanical insulted groups of 0.3 MPa (D–F), 0.5 MPa (G–I) and 0.7 MPa (J–L) presented obvious increased TUNEL positive cells which indicated apoptosis cells. The white arrows show the TUNEL positive cells. Scale bar, 50 μm.

**Fig. 7.**
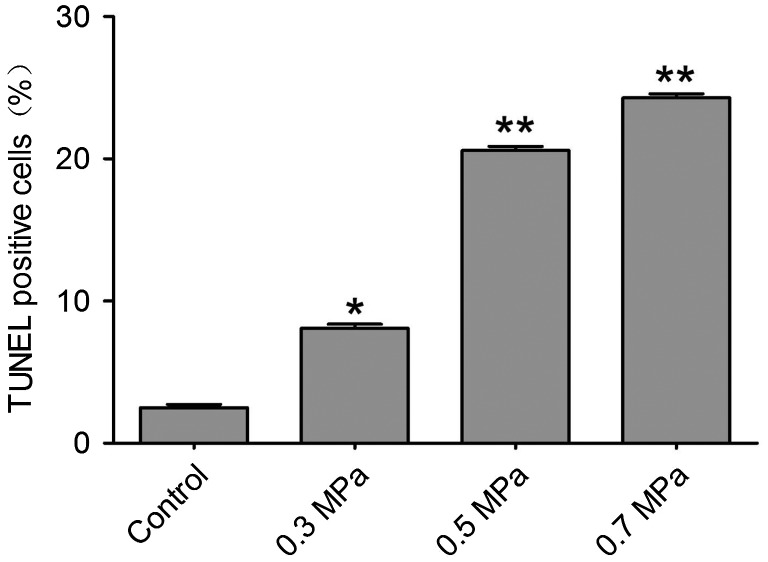
Quantification of TUNEL positive cells ascertained from apoptosis detection for various cell samples as indicated in Fig. 6. Notes: The bar graph represents the mean percentage (±SEM) of apoptosis cells in each condition. Samples with mechanical compression have a significant larger scale of apoptosis than their unstimulated controls (*p < 0.05, **p < 0.01).

Cell necrosis can be found by using Flow Cytometry in our preliminary experiment. In the mechanical compression groups, the percentage of necrosis cells increased significantly compared with the control group. The quantification of necrosis cells is showed in Fig. 5. The characteristic of apoptosis also can be found in our preliminary experiment by transmission electron microscope technology (Supplemental Fig. 2). The typical characteristic of apoptosis can be found by transmission electron microscope. Cytoplasmic vacuolation and chromatin condensation can be found (Supplemental Fig. 2). In addition, the nucleus membrane was unclear in the transmission electron microscope results (Supplemental Fig. 2). TUNEL positivity was observed in both the control group and the mechanical compression groups. TUNEL-positive DRG neurons were occasionally found in the control group; however, in the mechanical compression groups, the percentage of TUNEL-positive cells was significantly increased. Especially in the 0.3 and 0.5 MPa groups, the TUNEL-positive cell count increased significantly and was correlated with the increase in the mechanical compression value (Fig. 6). In the 0.7 MPa group, the number of TUNEL-positive cells was much higher than in the other groups, indicating that higher levels of mechanical compression may exceed the level that DRG neurons can tolerate, inducing more cell apoptosis. The quantification of TUNEL-positive cells (Fig. 7) for various cell samples indicated that mechanical compression induced significantly more apoptosis compared with their unstimulated counterparts. By using a spectrophotometric assay, we detected the activities of caspase-3 showed in Supplemental Fig. 3, Biosci. Biotech. Biochem. Web Site. A statistically significant increasing of activity of caspase-3 can be found following mechanical compression insults.

## Discussion

In the present study, we investigated the effects of mechanical compression on neurons at the ultrastructural level. We examined the nanoscale changes in the surface topography of DRG neurons in response to mechanical strain, and we also evaluated neuronal apoptosis and necrosis. Using AFM, we investigated the changes in the topological details of cultured DRG neurons following the application of mechanical compression force. These forces induced increased density and size of granular structures on the cell surface. We quantitatively analyzed the membrane particle structure and showed that the granular structures on the cell membrane were correlated with the change in the mechanical compression value. In addition, mechanical compression caused remodeling of the membrane skeleton, apoptosis, and necrosis. To the best of our knowledge, this report is the first to elucidate qualitative and quantitative changes in the surface topology of DRG neurons after mechanical compression, including compression-induced cytoskeletal remodeling, and the influence of mechanical force on cell survival through membrane-skeleton changes.

The pathophysiology of SCI is a complex process that is initiated by a primary mechanical injury and followed by a cascade of events termed secondary injury. The pathophysiology of SCI has traditionally been extensively studied *in vivo* in the intact animal.^19^ ^)^ Neuroscientists have attempted to reproduce the biomechanical environment during SCI using *in vitro* injury systems with isolated components of the nervous system. Thus, using this self-designed equipment, which has the ability to precisely and homogeneously deliver mechanical compression, we exclusively studied the responses of neuronal cells to mechanical compression injury. Excess mechanical force and deformation causes structural and functional breakdown, including several key deleterious cellular processes, such as membrane damage, disruption of calcium homeostasis, glutamate release, cell death, and caspase-mediated proteolysis.^20^ ^)^ However, little information is known about the ultrastructure of the cell membrane in the context of mechanical compression. Our present study provides detailed information of the neuronal cell membrane ultrastructure in response to the primary phase of CSI.

Forty years ago, Singer and Nicolson described the PM as having a “fluid mosaic” environment that randomly partitions proteins and lipids to achieve the lowest free energy.^21^ ^)^ Later evidence demonstrated that this partitioning of proteins is not homogenous and random but instead consists of clusters of structural proteins, enzymes, signaling receptors, transporters, and channels within lipid domains.^22,23^ ^)^ These membrane domains and their unique protein and lipid contents are important for many cellular functions. Membrane lipids are known to be associated with cell death including apoptosis and necrosis. These microdomains have been reported to act as molecular platforms that spatially organize membrane receptor molecules such as epidermal growth factor receptor and CD95 (Fas).^24^ ^)^ This reorganization is of crucial importance in the initiation and regulation of inflammatory processes and apoptosis. In present study, we discovered the correlation of nanoscale changes of PM and neuronal cell apoptosis. These may indicate the underlying mechanism of signaling molecules on cell membrane during apoptosis. In addition, our results agreed with previous study^25,26^ ^)^ which indicate that the regulation of membrane lipids levels in lipid rafts is one of the key factors of apoptotic and necrotic signal in cells. In mechanical compression circumstance, PM were damaged, termed mechanoporation, and this can significantly affect cell integrity and function, leading to disruptions of ion homeostasis, electrical activity, and cell signaling.^20^ ^)^ Nanoscale images and topographical investigations of the cell membrane after mechanical injury can help reveal the changes in these structural clusters within the cell membrane. As the results showed, mechanoporation phenomena could be observed in the 0.7 MPa group, which displayed a “pore canal” structure. Apoptosis and necrosis were detected in mechanical compression groups coupled with changed distribution of membrane particles. These structural changes may correspond to the biological changes in the cell membrane-skeleton.

The cytoskeleton is a network of protein filaments extending through the cell cytoplasm of eukaryotic cells. The cytoskeleton contains fundamental components in control of many aspects of cell behavior, movement, and metabolism, including proliferation, intracellular signaling, movement, and mechanotransduction. Interactions between the membrane skeleton and cytoskeletal components can contribute to the regulation of the “fluid mosaic” membrane and cytoskeletal dynamics.^27^ ^)^ Although the association between cytoskeletal components and the membrane skeleton had been previously described,^28^ ^)^ recent evidence has extended the new notion that cytoskeletal components can localize to the membrane and serve as platforms for cytoskeletal anchoring and for communication to the extracellular matrix via integrins, cadherins, occludins, and other cellular adhesion molecules. The transient anchoring of actin and other cytoskeletal elements to lipids on the cell membrane creates a skeleton meshwork.^29^ ^)^ The combination of membrane bilayer lipids and membrane proteins, along with their interaction with the cytoskeleton, can contribute to mechanotransduction and other critical physiological processes. Furthermore, cytoskeletal changes have been extensively reported in apoptotic cells in which alterations in cell shape and anchorage are dependent on reorganization of actin filaments and focal adhesion contacts.^30,31^ ^)^ Our findings in the present study agree with this theory. We found that the cell membrane topography alterations accompanied by the cytoskeletal remodeling when apoptosis and necrosis occur, which indicated that interactions of the cell membrane with the cytoskeleton, and perhaps their cooperative function of signal transduction, could lead to apoptosis and necrosis in response to mechanical stimulation.

AFM topographic imaging and surface roughness measurements have served as a valuable tool to quantify the distribution of specific membrane proteins on the cell surface. Although the biochemical changes of DRG neurons subjected to mechanical compression were not widely investigated, the results of compression on other cells, such as chondrocytes, indicated that the expression of a number of cell-surface receptors, such as the urokinase-type plasminogen activator receptor and membrane-bound matrix metalloproteases, increased.^32,33^ ^)^ Thus, the increase in the size of the granules observed by AFM on our mechanical compression-treated DRG neuron surface compared with the native cells could be explained by the increased expression of the above cell-surface receptors and/or their complexes. In our study, the 0.7 MPa group showed decreased particle size and density, suggesting that the 0.7 MPa mechanical compression exceeded the threshold of the DRG neurons and induced the degradation of membrane skeleton proteins. Our findings, for the first time, demonstrate a quantitative analysis of the distribution of cell-surface molecules in response to mechanical compression, which may provide new insights into the response of DRG neurons to mechanical factors. Nevertheless, there are many more questions to answer. Our future work involving the specific labeling of cell-surface molecules coupled with AFM analysis would help further ascertain the relative quantities and expression of these surface complexes.

In conclusion, mechanical compression can cause rearrangement of the membrane skeleton and cytoskeleton. DRG neurons have their own threshold to undergo mechanical stimulation. Our current study revealed the nanoscale response to mechanical compression on the cell membrane and the different biological responses after exposure to mechanical insult. These results may help in the search to further understand the mechanism of primary SCI and to identify protective agents for SCI.

## Author contribution

Conceived and designed the experiments: Xin Quan, Kai Guo, Zhuojing Luo. Performed the experiments: Xin Quan, Kai Guo, Yuqing Wang. Analyzed the data: Xin Quan, Kai Guo. Contributed reagents/materials/analysis tools: Liangliang Huang, Beiyu Chen. Wrote the paper: Xin Quan and Zhengxu Ye.

## Supplemental material

The supplemental material for this paper is available at http://dx.doi.org/10.1080/09168451.2014.932664.
